# Morphological characteristics of femoral neck fractures in young and middle-aged population: a retrospective descriptive study

**DOI:** 10.1186/s12891-024-07207-5

**Published:** 2024-01-29

**Authors:** Yingzhe Jin, Bohao Yin, Linyuan Shu, Zhiyuan Fan, Matthew C. Sherrier, Chenjun Liu, Hui Sun, Wei Zhang

**Affiliations:** 1 Orthopedic Department, The Affiliated Lihuili Hospital of Ningbo University, 1111 JiangNan Road, Ningbo, 315040 China; 2 Department of Orthopaedic Surgery, Shanghai Sixth People’s Hospital Affiliated to Shanghai Jiao Tong University School of Medicine, 600 YiShan Road, Shanghai, 200233 China; 3 Department of Emergency Medicine, Shanghai Sixth People’s Hospital Affiliated to Shanghai Jiao Tong University School of Medicine, 600 YiShan Road, Shanghai, 200233 China; 4 Department of Orthopaedics and Physical Medicine, Medical University of South Carolina, Charleston, SC 29425 USA

**Keywords:** Femoral neck fracture, Postoperative CT, Intraosseous bone defects, Classification, Morphology

## Abstract

**Background:**

A understanding of morphological characteristics are important to femoral neck fractures (FNFs) resulting in high rates of complications in the young and middle-aged adults and the detailed data is lack in the literature. We aimed to report on the detailed morphological characteristics and the relationship between them in young and middle-aged adults with femoral neck fractures (FNFs).

**Methods:**

The postoperative CT images of one hundred and fifty-two adults with FNFs were retrospectively reviewed. After image standardization, morphological characteristics including fracture orientation, cortex comminution, and intraosseous bone defects were measured and analyzed. Additionally, the distribution and correlation of these morphological features were analyzed using Pauwels classification, the right angle of the neck axis (VNA) classification, and the anteromedial oblique angle (AMA).

**Results:**

Pauwels III fractures accounted for approximately half (55.2%) of the FNFs analyzed. Pauwels II and III could be detected in all four VNA types, and the distribution of the Pauwels types in VNA classification showed significant differences (χ^2^ = 106.363, *p* < 0.001). The VNA (9.0° ± 12.1) showed positive correlation with the neck-shaft angle (139.5° ± 6.3) and modified Pauwels angle (49.8° ± 10.6) (*r* = 0.441, *r* = 0.855, all *p* < 0.001). Cortical comminutions were commonly observed in the posterior (86.7%) and the inferior (80.7%). AMAs within the cases without posterior and inferior cortex comminutions were significantly larger than those with comminution (t = 2.594, 2.1196; *p* = 0.01, 0.036), but no difference could be detected after the AMA being divided into three groups (< 85°, 85°-95°, > 95°). The MPA, VNA and AMA of the group with an intraosseous defect were significantly different compared with those without (t = 2.847, 2.314, 2.268; *p* = 0.005, 0.022,0.025). The incidence of intraosseous defects within the groups with coronal and axial cortex comminutions were significantly higher than those within the groups without comminutions (χ^2^ = 34.87, 25.303; *p* < 0.001).

**Conclusions:**

The present study highlights the morphological diversity and complexity within FNFs in young and middle-aged adults, which allows for more accurate simulation of FNF patterns in the future biomechanical studies.

**Supplementary Information:**

The online version contains supplementary material available at 10.1186/s12891-024-07207-5.

## Background

Femoral neck fractures (FNFs) are commonly encountered in orthopaedic practice, accounting for 3.6% of all fractures and 48%-54% of hip fractures [1]. Although occurring at a lower incidence than in the elderly, FNFs in the young and middle-aged adult population present a unique clinical challenge as most are secondary to high energy trauma and patients have higher post-operative functional demands for work or recreational activities. These fractures are typically complex in nature, with significant displacement, oblique fracture angles, and comminution [2]. It is known that these characteristics, along with disruption of the vascular supply, result in high rates of complications [3], including fracture failure, nonunion, and avascular necrosis of the femoral head [4–6]. Although many biomechanical and clinical studies have evaluated various options, the optimal fixation construct to allow for healing and prevention of complications after FNF is still unknown [7].

A better understanding of FNF morphology would provide an essential foundation for optimal biomechanical models and surgical treatment. However, few studies have focused on the detailed morphological characteristics of FNFs and the majority of them have concentrated on Pauwels type III fractures [2, 8, 9]. As such, the morphology of different types of FNFs experienced in clinical practice and the differences among them have not been well described. Thus, the objective of this study was to provide detailed morphological characteristics of FNFs in a young population based on postoperative computed tomography (CT) images and the relationship between them, such as fracture angles and cortical comminutions, in different FNF classifications.

## Methods

This study was a retrospective evaluation of radiographic images from patients in a level I trauma center from January 2020 to September 2021. The inclusion criteria were: (1) age ≤ 59 years, and older than 18 years; (2) no history of hip arthritis; (3) no morphological change of both hip joints or skeleton (e.g. congenital malformation, infantile paralysis, dysplasia); (4) who required closed reduction internal fixation surgery within two days after suffered an FNF (5) adequate radiological images, including postoperative radiography and CT images from the first day after surgery. Exclusion criteria included: skeletal immaturity; spinal scoliosis and pelvis inclination; pathological changes to the bone of the proximal femur such as periosteal hyperplasia, hyperostosis, or osteolysis; fragility or pathological fracture of femoral neck; accompanying fractures of the ipsilateral femoral head or shaft, trochanter region, pelvis, or acetabulum; patient underwent hip arthroplasty surgery, repeated or revision surgery for FNF treatment; a fair or poor reduction after internal fixation of FNF according to the standards of Haidukewych et al. [10]; lack of perioperative radiographs; CT scan slice thickness greater than 3 mm; blurred or distorted radiological images.

Due to the lack in consensus on grading the quality of FNF reduction, fracture reduction was graded on the amount of displacement and degree of residual angulation on immediate postoperative radiographs [10]. An excellent reduction is considered less than 2 mm of displacement and less than 5° of angulation in any plane; good reduction is 2–5 mm of displacement and/or 5°-10° of angulation; the displacement greater than 5 mm and angulation greater than 10° is considered fair or poor. The rate of fair and poor reduction in FNFs is less than 5% at our institution. The screening process was done by one radiologist and one orthopaedic surgeon. Any disagreements were resolved through negotiations.

### Radiographic preparation

CT imaging scans in neutral/supine were operated in all patients. Thin slice CT images were obtained using picture archiving and communication system workstations, and image screenings were performed using Kingstar Winning TV view software (Shanghai Kingstar Winning Medical Information Technology Co. Ltd., Shanghai, China), which has precision of 0.01 mm. The CT scan slice thickness used in this study was no more than 1 mm. The DICOM (Digital Imaging and Communication in Medicine) images were loaded and standardized using RadiAnt DICOM Viewer software (Version 2020.2.3, 64-bit, Medixant, Poland). For standardization, all CT images were changed to “bone window” and the “Multiplana reconstruction” function was opened. The coronal axis on the horizontal plane was then rotated to symmetrize the pelvis in the coronal plane and the coronal axis on the sagittal plane was rotated to symmetrize the pelvis in the horizontal plane (Supplement 1). After standardization, the reconstructed images were saved and imported into the Mimics Medical software (version 21.0) for subsequent measurement. 3-D models of the proximal femur were reconstructed using threshold segmentation and interactive editing method in the Mimics software.

All imaging assessments were conducted by two authors who were blinded each other, including the patients’ demographics and the measurement calculated by other assessor. Each assessor performed their assessment twice to reduce inter- and intra-observer variations.

### Morphological assessment and measurement

**Fig. 1:**
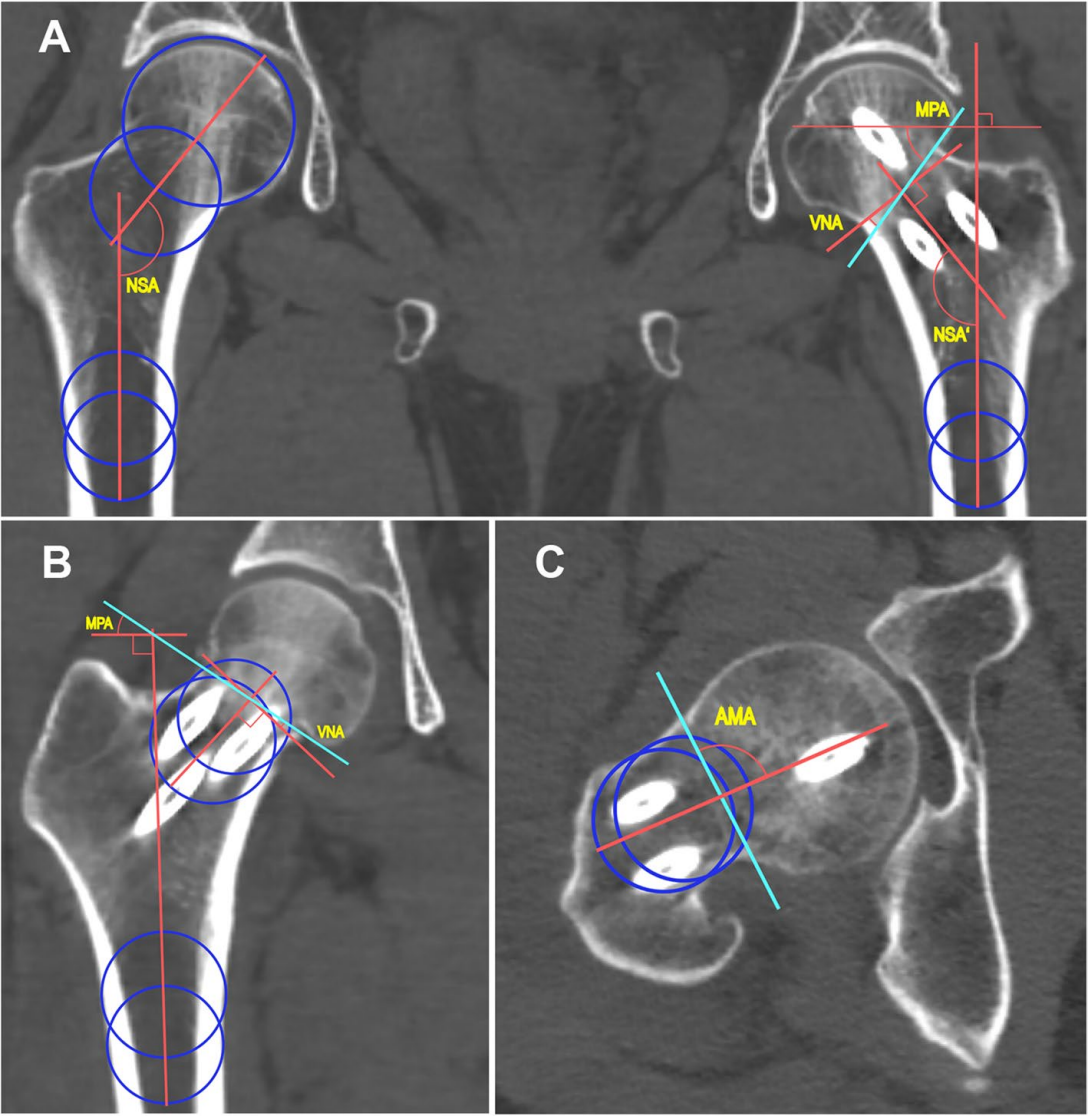
Radiographic measurements of angle parameters in coronal and axial planes. Two circles (blue) tangent to the lateral cortex of the femoral shaft and neck were drawn in each plane. The lines connecting the center points of two circles were the centerlines (red) of the femoral shaft and neck. The perpendicular lines of the centerlines were also red. The fracture lines were labeled as washed blue. **A**. Indirect measurement method of MPA and VNA. **B**. Direct measurement method of MPA and VNA. **C**. Measurement method of AMA

Image slices with the widest femoral neck view in the coronal and axial planes were selected for evaluation. The centerlines of the femoral shaft and neck on the coronal and axial planes were determined by drawing two circles tangent to the lateral cortex of the femoral shaft and femoral neck in each plane and forming a connecting line to the center points of the two circles (Fig. 1). In order to confirm whether this method (the circumscribed circle method) could be used to our study, NSA was measured by three observers separately on the CT image data of 50 patients randomly numbered twice a month. The inter- and intra- observer reliability of this method were showed in Supplement with changes marked, which demonstrated that this method could be used to our study. The main fracture lines were defined as the lines connecting the broken ends of the superior/inferior and anterior/posterior cortexes of the distal shaft-neck fragment in the coronal and axial planes, respectively.

All imaging assessments were conducted by two authors who were blinded each other, including the patients’ demographics and the measurement calculated by other assessor. Each assessor performed their assessment twice to reduce inter- and intra-observer variations and the mean value of their measurements is the final result.

#### Angle parameters on the coronal plane and classification

The angle parameters included the neck-shaft angle (NSA), the modified Pauwels angle (MPA) and the right angle of the neck axis (VNA) (Fig. 1 A and B).

The NSA is defined as the angle between the centerlines of the femoral shaft and neck [11]. The MPA is defined as the angle between the fracture line and an imaginary line perpendicular to the centerline of femoral shaft [12]. Using this method, the FNFs were classified into three types according the Pauwels classification: type I, < 30°; type II, 30–50°; and type III, > 50°. The VNA is the angle between the fracture line and the vertical line of the neck axis [13]. According to a prior study, the fractures were divided into four VNA types: type I < 0°; type II 0°-10°; type III 10°-15°; type IV ≥ 15°. A positive angle is defined as a more vertical fracture line. The larger value means greater shear force acts to fragments after surgery. In most cases, these angles could be measured directly on the images of injured side (direct measuring method, *n* = 137). In cases with insufficient neck length, the femoral neck axis was labelled according to the NSA of the uninjured femur and the vertical line was fixed (indirect measuring method, *n* = 15) [13]. Each fracture was also described based on the anatomic location.

#### Angle parameters on the axial plane and grouping

The anteromedial oblique angle (AMA) is defined as the angle formed by the fracture line crossed with the centerline of femoral neck in the axial plane (Fig. 1C). The AMA was divided into three groups: Group I, < 85° (posterior subtype); Group II, 85°-95° (classical subtype) and Group III, > 95° (anterior subtype) [8].

#### Cortical comminution and intraosseous defects

**Fig. 2:**
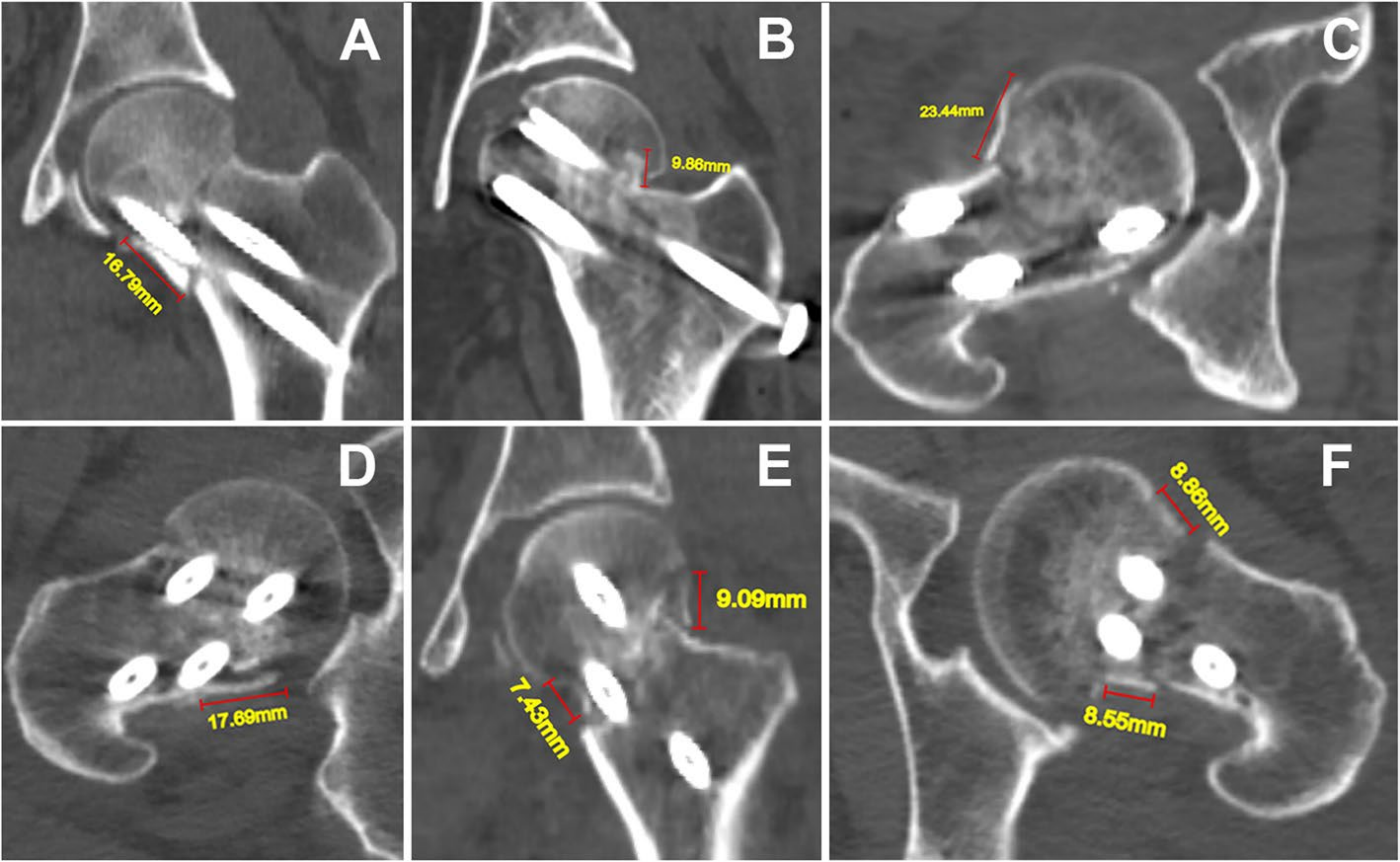
Radiographic measurements of cortical comminution in coronal and axial planes. **A** and **B**. Isolated comminutions of the inferior and superior cortex. **C** and **D**. Unilateral comminutions of the anterior and posterior cortex. **E**. Combined comminution of both inferior and superior cortices. **F**. Bilateral comminution of both anterior and posterior cortices

**Fig. 3:**
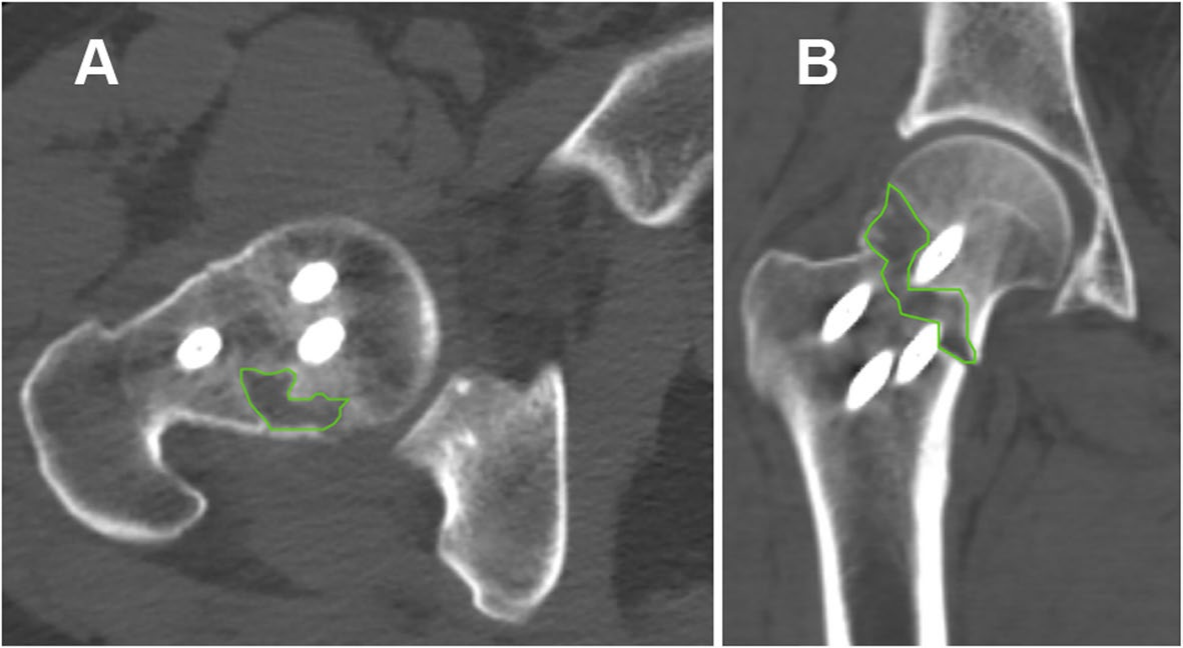
Representative intraosseous bone defects in axial (**A**) and coronal (**B**) planes. Outlines of the largest intraosseous defect area were sketched using green lines

Cortex comminution is defined by the presence of separate fragments of any size and dimension that are not continuous between the head-neck and shaft-neck fragments [9]. The parameters of cortical comminutions included location and maximum length in the coronal and axial planes. Location was described by position of the comminution observed in the coronal and axial planes (inferior, superior, anterior and posterior positions). The length of cortical comminutions was measured on CT slices in which the fragment was the longest (Fig. 2). The intraosseous bone defect was depicted by a high transmission area with clear boundaries within the cancellous bone of the femoral neck. The bone defect was outlined in both coronal and axial planes where the area of the defect was largest (Fig. 3).

### Statistical analysis

Sample size simulated by an independent statistician using “one mean” function of PASS 11.0 (PASS, NCSS, LLC), was the number of 111 patients lower than a total of 152 patients in our study. Statistical analyses were performed by an independent statistician using SPSS 26.0 (SPSS, Inc., Chicago, IL, US). Continuous variables with normal distribution were presented as mean ± SD and tested by independent-sample t test or analysis of variance (ANOVA). Data with skewed distributions were presented as median (interquartile range) and tested by Mann–Whitney U test or Cruskal-Wallis H test. Categorical variables were shown as frequency and percentages (%) and tested by chi-squared test (χ2), including Pearson correlation or Fisher’s exact test. Pearson or Spearman correlation test were used for correlation analyses between variables. A value of *p* < 0.05 indicated statistical significance.

## Results

A total of 152 patients with unilateral FNFs met the inclusion criteria for this study. Patient demographics and fracture characteristics are summarized in Table 1.

**Table 1:** Subject demographics and fracture characteristics (*n* = 152)

Variables	Value	
Patient demographics		
Age (yrs, range)	48 ± 10.9(18—59)	
Sex (Male n, %)	78 (51.3%)	
Side (Left n, %)	73 (48%)	
BMI (Kg/m^2^, range)	22.8 ± 3.4 (15.0—33.8)	
Underweight (< 18.5, %)	13 (8.7%)	
Normal (18.5 ― 24.9, %)	101 (67.8%)	
Overweight (25 ― 29.9, %)	32 (21.5%)	
Obesity (> 30, %)	3 (2.0%)	
NSA (, range)	139.5 ± 6.3 (125.5—161.8)	
Fracture characteristics		
Anatomical classification (n, %)		
Subcapital	23 (15.1%)	
Transcervical	125 (82.3%)	
Basicervical	4 (2.6%)	
MPA (°, range)	49.8 ± 10.6 (25.6 ― 76.0)	
Pauwels classification (n, %) ― (°, range)		
Type I	8 (5.3%)	27.8 ± 1.9 (25.6—30.0)
Type II	60 (39.5%)	42.1 ± 5.4 (30.5—49.9)
Type III	84 (55.2%)	57.3 ± 6.0 (50.1—76.0)
VNA (°, range)	9.0 ± 12.1 (-24.8 ― 38.1)	
VN classification (n, %) ― (°, range)		
Type I	35 (23.0%)	-7.2 ± 5.9 (-24.8—0.02)
Type II	42 (27.6%)	4.9 ± 2.8 (0.2—9.3)
Type III	28 (18.4%)	12.4 ± 1.6 (10.0—14.6)
Type IV	47 (31.0%)	22.6 ± 6.0 (15.1—38.1)
AMA (°, range)	93.1 ± 13.7 (46.6 ― 130.5)	
AMA groups (n, %) ― (°, range)		
Group I	34 (22.4%)	75.2 ± 9.5 (46.6—84.6)
Group II	54 (35.5%)	90.3 ± 3.0 (85.0—95.0)
Group III	64 (42.1%)	105.0 ± 8.5 (95.2—130.5)
Cortex comminution in coronal plane (n, %)	114 (75%)	
Location (n, %) ― Length (mm, range)		
Superior	64 (56.1%)	11.2 ± 4.1 (2.56—22)
Inferior	92 (80.7%)	13.2 ± 5.4 (3.96—27.9)
Cortex comminution in axial plane (n, %)	120 (78.9%)	
Location (n, %) ― Length (mm, range)		
Anterior	49 (40.8%)	10.1 ± 4.7 (4.16—28.7)
Posterior	104 (86.7%)	13.8 ± 4.5 (1.63—28.5)
Intraosseous defects (n, %)	79 (52.0%)	

*BMI* Body mass index, three patients' BMI was lost. *NSA* Neck-shaft angle, *MPA* Modified Pauwels angle, *VNA* Angle of vertical of the neck axis, *AMA* Anteromedial angle. Continuous variables with normal distribution were presented as mean ± SD (range). Categorical variables were shown as number and percentages (%)

### Relationship among different classifications and angles

**Fig. 4:**
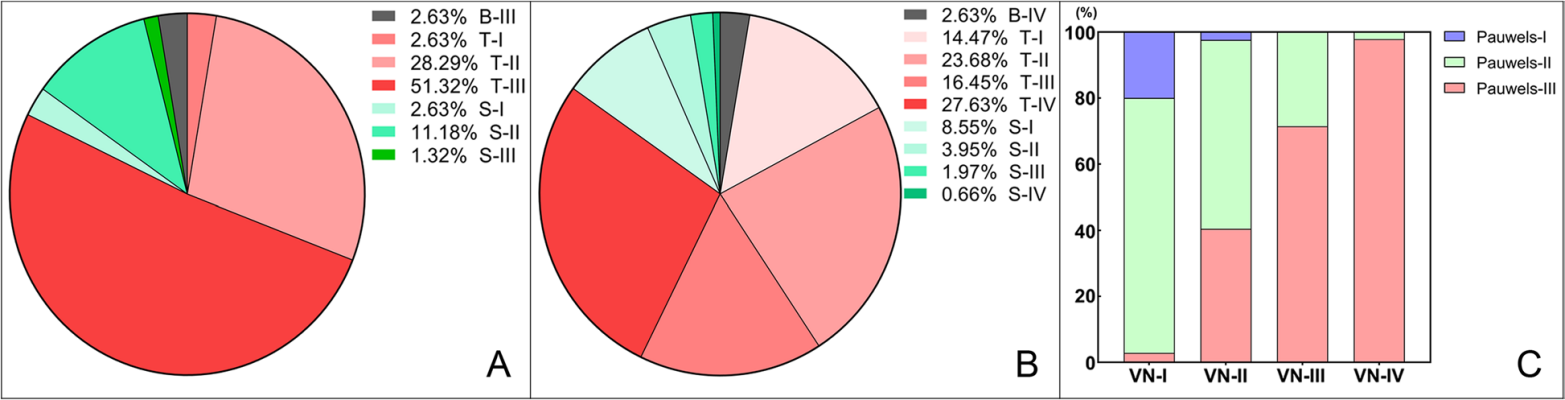
The distributions of fractures by compound classifications. **A**. Anatomical-Pauwels compound classification. **B**. Anatomical-VN compound classification. **C**. Pauwels classification crossing with VN classification. S, Subcapital type; T, Transcervical type; B, Basicervical type

**Fig. 5:**
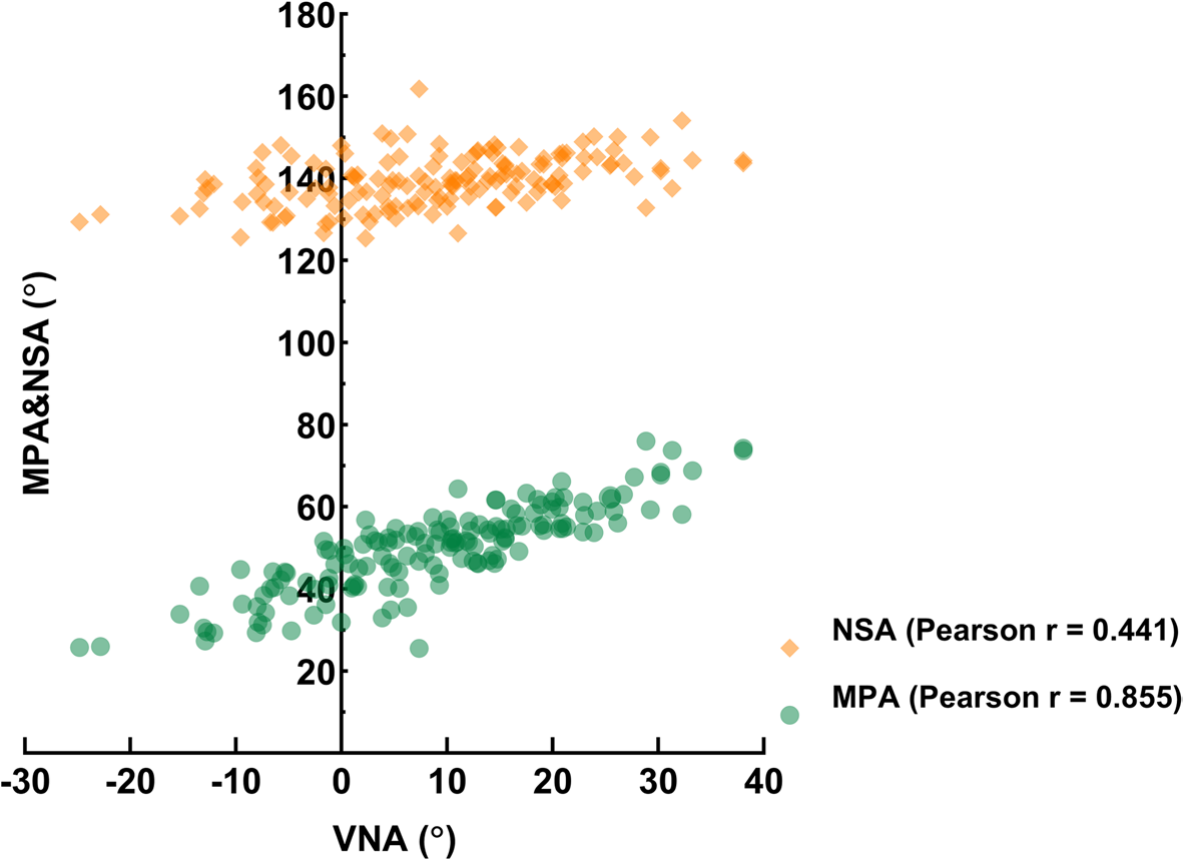
Correlation analyses between the VNA and NSA, the VNA and MPA

Fractures were most commonly transcervical (82.3%) and 55.2% were Pauwels type III (Table 1). According to the compound classifications, the most common type of FNF observed was the transcervical-Pauwels type III (51.32%) (Fig. 4A) and the transcervical-VNA type IV (27.63%) (Fig. 4B). After crossing the Pauwels types with VNA types, the Pauwels type II and III could be detected in all four VNA types (Fig. 4C), and the distribution of the Pauwels types in VNA classification showed significant differences (χ2 = 106.363, *p* < 0.001). Figure 5 shows the positive correlation between the VNA and NSA (*r* = 0.441, *p* < 0.001) and the positive correlation between the VNA and MPA (*r* = 0.855, *p* < 0.001), indicating that the greater NSA and MPA, the more severe VNA. There was no correlation of the MPA with NSA (*r* = -0.059, *p* = 0.468).

### Distributions and length’s characteristics of cortical comminutions on both the coronal and axial planes

Cortical comminutions were commonly observed in both the coronal (75%) and axial planes (78.9%). In the coronal plane, comminuted fractures were more commonly observed in the inferior cortex (80.7%) when compared with the superior cortex (56.1%) (χ^2^ = 15.9, *p* < 0.001). The inferior cortex comminution length was significantly longer than that of the superior cortex (t = 2.731, *p* = 0.007). The length of superior cortex comminution had moderate positive correlation with the length of inferior cortex comminution (*r* = 0.454, *p* = 0.002). In the axial plane, comminuted fractures were more frequently noted in the posterior cortex (86.7%) when compared with the anterior cortex (40.8%) (χ^2^ = 54.5, *p* < 0.001). The posterior cortex comminution length was significantly longer than that of the anterior cortex (t = 4.719, *p* < 0.001). Correlation between the length of anterior cortex comminution and the posterior length was not statistically significant (*r* = -0.091, *p* = 0.614).

### Distributions and length’s characteristics of cortical comminutions among cortical comminution and classifications of Pauwels and VNA

**Fig. 6:**
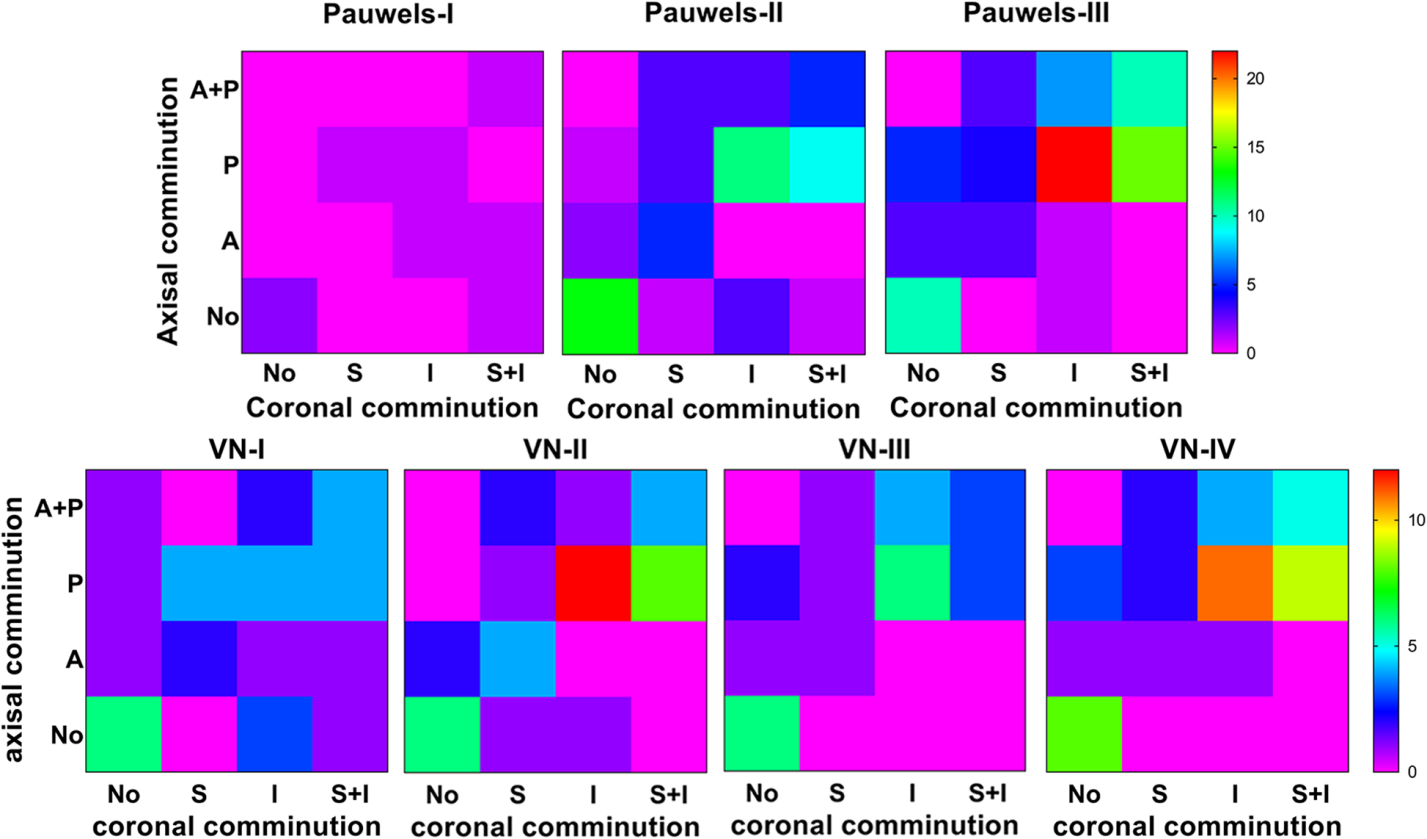
Heatmaps demonstrating the comprehensive distribution of cortical comminution among Pauwels and VN classifications. A, Anterior; P, Posterior; S, Superior; I, Inferior. A + P, Comminutions coexisting in both anterior and posterior cortices. S + I, Comminutions coexisting in both superior and inferior cortices

There were no differences among the three Pauwels types and four VNA types with respect to cortical comminution in the coronal plane (Table 2). Among the three Pauwels types, the distribution of posterior cortex comminution demonstrated significantly higher incidence in Pauwels type III fractures (77.4%, p = 0.027). Of note, there were posterior cortex comminutions seen in in Pauwels type I and II fractures. The distribution of posterior cortex comminution among the four VNA types exhibited no differences (*p* = 0.297). The length of posterior cortex comminution among three Pauwels types (*p* = 0.028) and four VNA types (*p* = 0.005) all demonstrated significant differences with longer lengths seen in increasing fracture severity (Table 2). A comprehensive distribution of cortical comminution in both the coronal and axial planes is shown in Fig. 6.

**Table 2:** Distribution and length of cortical comminution

Variables	Cortical comminution in coronal plane				Cortical comminution in axial plane			
	Superior (n, %)	Superior length (mm)	Inferior (n, %)	Inferior length (mm)	Anterior (n, %)	Anterior length (mm)	Posterior (n, %)	Posterior length (mm)
Pauwels classification								
Type I (8)	3 (37.5)	12.63 ± 4.83	4 (50.0)	14.78 ± 10.02	4 (50)	13.54 ± 6.19	4 (50)	10.25 ± 4.04
Type II (60)	26 (43.3)	10.66 ± 4.53	33 (55.0)	11.61 ± 5.24	18 (30)	9.00 ± 3.61	35 (58.3)	12.67 ± 3.77
Type III (84)	35 (41.7)	11.40 ± 3.69	55 (65.5)	14.09 ± 4.94	27 (32.1)	10.33 ± 4.94	65 (77.4)	14.66 ± 4.69
F/2	0.114	0.446	1.995	2.479	1.293	1.671	7.203	3.716
p	0.945^a^	0.642	0.369^a^	0.090	0.524^a^	0.199	0.027^a^	0.028
VN classification								
Type I (35)	16 (45.7)	11.39 ± 4.64	20 (57.1)	10.73 ± 6.05	12 (34.3)	10.11 ± 4.84	20 (57.1)	11.53 ± 3.36
Type II (42)	20 (47.6)	10.93 ± 4.49	26 (61.9)	13.15 ± 5.14	13 (31.0)	9.83 ± 3.35	28 (66.7)	12.73 ± 4.25
Type III (28)	9 (32.1)	11.66 ± 4.60	16 (57.1)	14.24 ± 5.25	10 (35.7)	11.89 ± 6.93	20 (71.4)	14.72 ± 4.45
Type IV (47)	19(40.4)	10.96 ± 2.97	30 (63.8)	14.35 ± 4.86	14 (29.8)	9.06 ± 3.60	36 (76.6)	15.44 ± 4.50
F/2	1.905	0.096	0.550	2.150	0.383	0.724	3.691	4.505
p	0.592	0.962	0.908	0.100	0.994	0.543	0.297	0.005

^a^Fisher’s exact test

### Relationship among the AMA, other angles, and cortex comminutions

There were no correlations among the AMA, NSA, MPA, and VNA (*r* = -0.100, -0.004, -0.061, *p* = 0.218, 0.958, 0.454). There was also no correlation observed between the AMA and the anterior and posterior cortex comminution lengths (*r* = -0.073, -0.042, *p* = 0.620, 0.669). There was a negative weak correlation between the AMA and the rate of posterior cortex comminution (*r* = -0.184, *p* = 0.023). The AMAs within the cases without posterior and inferior cortex comminutions were significantly larger than those with comminution (t = 2.594, 2.1196; *p* = 0.01, 0.036) (Table 3). No significant difference could be detected among the three AMA groups (Supplement 2).

**Table 3:** AMA comparison between fractures with and without cortical comminution in two planes

	AMA	t	p
Anterior comminution			
No (103)	93.12 ± 12.88	0.037	0.971
Yes (49)	93.04 ± 15.45		
Posterior comminution			
No (48)	97.27 ± 12.45	2.594	0.010
Yes (104)	91.18 ± 13.88		
Superior comminution			
No (88)	94.83 ± 13.78	1.84	0.068
Yes (64)	90.72 ± 13.33		
Inferior comminution			
No (60)	95.98 ± 13.77	2.119	0.036
Yes (92)	91.22 ± 13.40		

### Relationship among intraosseous defects groups, the three angles, two classifications and cortex comminutions

The overall incidence of intraosseous defects was 52.0%. The MPA and VNA of the group with an intraosseous defect were significantly higher than those without (t = 2.847, 2.314; *p* = 0.005, 0.022) (Table 4). Conversely, the AMA of the group with intraosseous defects was significantly lower than that of the group without (t = 2.268; *p* = 0.025) (Table 4). No significant difference was seen in the distribution of intraosseous defects among the three Pauwels types and four VNA types. The incidence of intraosseous defects within the groups with coronal and axial cortex comminutions were significantly higher than those within the groups without comminutions (χ^2^ = 34.87, 25.303; *p* < 0.001) (Table 4).

**Table 4:** The distribution of radiographic parameters and classifications between the groups with and without intraosseous defects

Variables	No intraosseous defect (73)	Intraosseous defect (79)	t/χ2	p
NSA (°)	139.9 ± 6.6	139.0 ± 5.9	0.824	0.411
MPA (°)	47.30 ± 10.25	52.08 ± 10.43	2.847	0.005
Pauwels classification (n, %)				
Type I (8)	6 (8.2)	2 (2.5)	3.643	0.162^a^
Type II (60)	31 (42.5)	29 (36.7)		
Type III (84)	36 (49.3)	48 (60.8)		
VNA (°)	6.62 ± 11.52	11.11 ± 12.35	2.314	0.022
VN classification (n, %)				
Type I (35)	21 (28.8)	14 (17.7)	2.877	0.411
Type II (42)	20 (27.4)	22 (27.8)		
Type III (28)	12 (16.4)	16 (20.3)		
Type IV (47)	20 (27.4)	27 (34.2)		
AMA (°) Coronal	95.69 ± 13.58	90.71 ± 13.46	2.268	0.025
Cortex comminution (114)	39 (34.21)	75 (65.79)	34.87	< 0.001
No cortex comminution (38) Axial	34(89.47)	4(10.53)		
Cortex comminution (120)	45 (37.50)	75 (62.50)	25.303	< 0.001
No cortex comminution (32)	28(87.50)	4(12.50)		

^a^Fisher’s exact test

### Distribution of the different measured parameters between two genders and others data

The distribution of NSA, MPA, VNA and AMA between two genders in all included patients are shown in Supplement 3 with females having significantly higher NSA and significantly lower MPA and VNA. There were also significant differences with respect to gender for anatomic classification, modified Pauwels types, and VNA classification (Supplement 4). There was no difference among the three AMA groups with respect to age, gender, or affected side (Supplement 4). There were significant differences with regards to MPA, VNA, Pauwels classification, and VN classification among the three anatomical fracture types (Supplement 5).

## Discussion

FNFs in young and middle-aged patients represent a difficult injury to manage due to high rates of treatment failure [3, 5]. The distinct mechanical features and complex periarticular soft tissue anatomy of the proximal femur creates a challenging biomechanical and biological environment for fracture healing [14–16]. Although many options exist, there is a lack of consensus on the optimal surgical treatment method [6, 16]. To improve the clinical results of FNF surgical fixation techniques in young adults, better understanding of the morphologic characteristics of FNF could be relevant. Our results demonstrate the morphological diversity and complexity of FNFs in young and middle-aged patients. Furthermore, our results indicate that the degree of cortical comminution, fracture angle in the axial plane, intraosseous bone defects, and the correlations among these factors may have been underestimated in previous studies [2, 8, 9, 17].

Three studies on the topic of FNF morphology have been published in the past decade [2, 8, 9]. These studies have been limited by small number of subjects [5, 10] and a limited focus on the impact of the axial obliquity of fracture on the biomechanical stability in Pauwels type III FNFs [8]. Furthermore, the methods available for the study of fracture morphological are limited, with existing techniques primarily based on preoperative radiographic images. However, it has been shown that analysis of preoperative CT images without standardization and image reconstruction is difficult and inaccurate [2]. To our knowledge, this is the first study to comprehensively describe and multidimensionally analyze the entire spectrum of the morphological features of FNFs in young and middle-aged adults with a relatively larger number of cases using postoperative CT images.

A review of the experimental biomechanical literature on FNF reveals significant variability in fracture angles and cortical comminution in dissimilar fracture models (Supplement 6), limiting the extrapolation of results to clinical practice. FNFs in young patients are typically a result of high-energy trauma and more frequently present as vertically oriented and unstable fractures that require internal fixation [16, 18]. As such, most studies have focused on Pauwels type III or vertical fractures. However, Pauwels type III fractures are not representative of all FNFs young and middle-aged patients experience in clinical practice [4]; in our study Pauwels type III fractures only account for 55.2% of FNFs. Additionally, the majority of fracture models have not examined cortical comminution which is not an uncommon feature of FNFs [2, 9] and is likely an important factor in influencing the structural stability. The results of the present study indicate a wide range and degree of cortical comminution. Thus, limitations of existing biomechanical studies are secondary to an incomplete understanding of the pathomorphology of FNFs, particularly when compared with other fractures [19, 20]. The current study fills an important knowledge gap in this domain.

The Pauwels classification is frequently utilized as a therapeutic guideline to determine the appropriate treatment for FNFs [21, 22]. However, clinical utility and reliability of this classification has been called into question [22–24]. Therefore, the VNA and classification has been proposed, which provides better validity and reliability in FNFs than the Pauwels classification [13]. Besides, with the increase of type of the VNA classification and the Pauwels classification, the rate of short-term complications, such as screw loosening, varus collapse, obvious fracture displacement and femoral neck shortening, is gradually increasing, but the former is proved to be more related to the rate [13]. However, whether the rate of long-term complications such as nonunion and avascular necrosis of femoral head correlates with Pauwels classification is Controversial [12, 23].To our knowledge, the correlation between VNA and classification and other morphological features have not been analyzed. In our study, we found that the distribution of Pauwels types in VNA classification showed significant differences. We also demonstrated a positive correlation of VNA with the NSA and MPA, whereas no correlation was shown between MPA and NSA. In addition, the distribution of cortical comminutions was gradually concentrated on both posterior and inferior cortices with the increase of VNA classification as shown by the heatmap, but Pauwels classification don’t have this trend. Thus, the VNA measurement provides a more nuanced classification than the Pauwels system, and may prove useful in facilitating stability assessment for the FNFs.

With regards to axial measurements, we present the first study to show the relationship of AMA to other morphological features. While no correlations could be detected between AMA and NSA, MPA, VNA, and the comminution lengths, we did discover that AMA is smaller in the cases with posterior and inferior comminution and those with intraosseous bone defects and has a weak negative correlation with the rate of posterior cortex comminution. This result is consistent with Collinge’s study [9]. The reason may be that the cases with posterior and inferior comminution suffer a more severe varus and internal rotational deformity compared with the cases without. The deformity more easily results in the appearance of posterior and inferior comminutions in the distal neck-shaft fragment which is longer in the posterior cortices and the inferior cortices and the compression of the intramedullary cancellous bone which forms the intraosseous bone defects when fracture fragment is reduced. In a biomechanical study, smaller AMA indicates lower torque compared to larger AMA. Which imply that AMA may be a useful predictor of axial stability [17, 25]. However, no significant morphological difference could be detected among the three AMA groups, which similarly means AMA classification founded by Wang [8] need to considerable again.

Cortex comminution is a common, yet underestimated, problem in the management of FNFs (Supplement 6). Although they can be detected in all quadrants of the femoral neck [9], femoral neck comminution in Pauwels type III fractures, are mostly seen in the inferior and posterior quadrants [2]. Consistent with existing literature, we found that comminution was more commonly seen in the inferior (80.7%) and posterior (86.7%) cortices, but also appears in the superior (56.1%) and anterior (40.8%) cortices, regardless of Pauwels or VNA classifications. For FNFs in young and middle-aged adults, posterior comminution has been recognized as a main contributory factor to result in complications, such as femoral neck shortening, nonunion and avascular necrosis of the femoral head [4, 17, 26–34]. However, previous studies about posterior comminution have been qualitative, that length of comminution have an adverse effect on clinical outcome is unknown. In our study, the length of posterior comminution is between 1.63 mm and 28.5 mm, the range of which is so large that the clinical outcomes must be different. Besides, the contribution of cortex comminutions in other quadrants and their correlations to other fracture features and the clinical outcomes have not been investigated.

Bone defects are relatively common in peri-articular, osteoporotic, or pathological fractures [35, 36]. It is generally recognized that intraosseous bone defects in FNFs are caused by compression of the intramedullary cancellous bone by the displaced fracture. However, intraosseous bone defects in FNFs have not been described to this point. The use of postoperative images in this study allow for the first description of these bone defects, although quantitative volumetric measurement of the intraosseous bone defect in the FNFs remains a challenge due to metal artifacts of the implants in our study [37]. We found that intraosseous bone defect group had larger both MPA and VNA and higher rate of cortex comminution which indicated intraosseous bone defect may have ramifications in fracture nonunion and inescapability of bone grafting, although the clinical implications of these intraosseous bone defects require further study.

The strengths of the present study include the large number of consecutive patients who had been treated at a single institution and the high availability of postoperative CT images. Limitations include exclusion of patients with fair and poor quality of fracture reduction, and ipsilateral femoral neck and shaft fractures. Additionally, although we have chosen the FNF patients with the surgery pattern which have a much smaller impact on the morphological characteristics during surgery, the retrospective nature of this study leads to the involvement of multiple surgeons with differing fixation techniques and devices, which may exert various degrees of impact on the morphological analysis. Finally, the reliability of the measurements of each morphological parameter has not been fully assessed.

## Conclusions

In conclusion, with a comprehensive understanding of the morphological characteristics of FNFs in young and middle-aged patients, further biomechanical experiments can be conducted that more accurately simulate injury patterns encountered clinically. The VNA classification exhibits more subtle discernibility than the Pauwels classification for FNFs. Our study examined several clinically important characteristics of FNFs in young adults, such as cortical comminutions, the oblique angle of fractures in axial plane and intraosseous bone defects. Overall, the results of the current analysis highlight the morphological diversity and complexity with young and middle-aged FNFs.

### Supplementary Information

**Additional file 1: Supplement.** ICC value for inter- and intra- observer for the circumscribed circle method to measure NSA. [ICC(95% IC)].

**Additional file 2: Supplement 1.** Coronal and axial CT images before and after standardization.** Supplement 2.** ICC value for inter- and intra- observer for the circumscribed circle method to measure NSA. [ICC(95% IC)].** Supplement 3.** The distribution of radiographic parameters and classifications among three AMA groups.** Supplement 4.** Gender differences with NSA, MPA, VNA and AMA.** Supplement 5.** The distribution of anatomical, Pauwels, and VNA classifications and AMA groups with respect to key demographic characteristics.** Supplement 6.** The distribution of MPA, VNA, Pauwels classification, and VNA classification among three anatomical fracture types.** Supplement 7.** Literature review from 2011-2022 of the morphological details of the fracture models in physical biomechanical studies focusing on FNFs in young population.

## Data Availability

The datasets used and/or analysed during the current study are available from the corresponding author on reasonable request.

## Abbreviations

VNA: The angle of vertical of the neck axis
MPA: Modified Pauwels angle
AMA: The anteromedial oblique angle
NSA: The neck-shaft angle
FNF: Femoral neck fracture
CT: Computed tomography
BMI: Body mass index
