# Supplementary material for: Morphological characteristics of femoral neck fractures in young and middle-aged population: a retrospective descriptive study
Source: BMC Musculoskelet Disord. 2024 Jan 29;25:100. doi: 10.1186/s12891-024-07207-5 (PMC10823608; doi:10.1186/s12891-024-07207-5)
Supplement: Supplementary file 1 — Additional file 1: Supplement. ICC value for inter- and intra- observer for the circumscribed circle method to measure NSA. [ICC(95% IC)]. [file 12891_2024_7207_MOESM1_ESM.docx]

**Supplement.** ICC value for inter- and intra- observer for the circumscribed circle method to measure NSA. [ICC（95% IC]

| Type | Interobserver | |  |  | | Intraobserver |
| --- | --- | --- | --- | --- | --- | --- |
|  | A | | B | C | |  |
| A | X |  | | |  | 0.89（0.78-0.93） |
| B | 0.91（0.83-0.96） | X | | |  | 0.83（0.70-0.90） |
| C | 0.89（0.74-0.95） | 0.91（0.80-0.96） | | | X | 0.88（0.78-0.93） |
