# Supplementary material for: Morphological characteristics of femoral neck fractures in young and middle-aged population: a retrospective descriptive study
Source: BMC Musculoskelet Disord. 2024 Jan 29;25:100. doi: 10.1186/s12891-024-07207-5 (PMC10823608; doi:10.1186/s12891-024-07207-5)
Supplement: Supplementary file 2 — Additional file 2: Supplement 1. Coronal and axial CT images before and after standardization. Supplement 2. ICC value for inter- and intra- observer for the circumscribed circle method to measure NSA. [ICC(95% IC)]. Supplement 3. The distribution of radiographic parameters and classifications among three AMA groups. Supplement 4. Gender differences with NSA, MPA, VNA and AMA. Supplement 5. The distribution of anatomical, Pauwels, and VNA classifications and AMA groups with respect to key demographic characteristics. Supplement 6. The distribution of MPA, VNA, Pauwels classification, and VNA classification among three anatomical fracture types. Supplement 7. Literature review from 2011-2022 of the morphological details of the fracture models in physical biomechanical studies focusing on FNFs in young population. [file 12891_2024_7207_MOESM2_ESM.docx]

**Supplement 1.** Coronal and axial CT images before and after standardization.


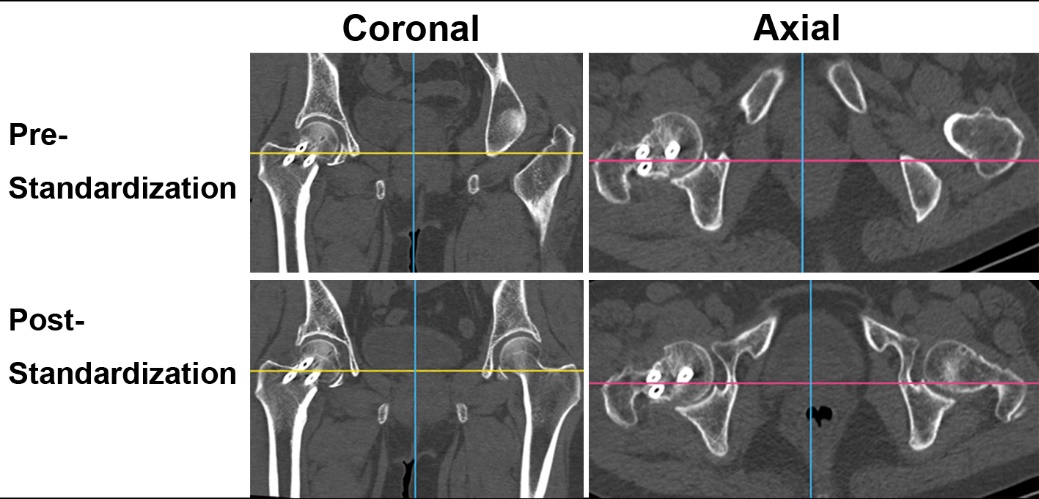


**Supplement 2**. ICC value for inter- and intra- observer for the circumscribed circle method to measure NSA. [ICC（95% IC）]

| Type | Interobserver | |  |  | | Intraobserver |
| --- | --- | --- | --- | --- | --- | --- |
|  | A | | B | C | |  |
| A | X |  | | |  | 0.89（0.78-0.93） |
| B | 0.91（0.83-0.96） | X | | |  | 0.83（0.70-0.90） |
| C | 0.89（0.74-0.95） | 0.91（0.80-0.96） | | | X | 0.88（0.78-0.93） |

**Supplement 3.** The distribution of radiographic parameters and classifications among three AMA groups.

| Variables | | Group I: AMA < 85° (34) | Group II: 85° ≤ AMA ≤ 95° (54) | Group III: AMA > 95° (64) | F/χ2 | p |
| --- | --- | --- | --- | --- | --- | --- |
| NSA (°) |  | 141.3 ± 6.7 (129.3 - 154.1) | 139.1 ± 6.7 (125.5 - 150.9) | 138.8 ± 5.5 (126.7 - 150.8) | 2.049 | 0.132 |
| PMA (°) |  | 50.0 ± 10.3 (25.6 - 66.2) | 50.2 ± 10.7 (25.8 - 74.3) | 49.4 ± 10.8 (26.0 - 76.0) | 0.084 | 0.919 |
| Pauwels classification (n) | | | | | | |
|  | Type I (8) | 1 (2.9) | 1 (1.9) | 6 (9.4) | 0.055 | 0.973 |
|  | Type II (60) | 14 (41.2) | 24 (44.4) | 22 (34.3) |  |  |
|  | Type III (84) | 19 (55.9) | 29 (53.7) | 36 (56.3) |  |  |
| VNA (°) |  | 11.0 ± 11.3 (-13.1 - 32.3) | 9.1 ± 12.9 (-24.8 - 38.1) | 7.7 ± 11.9 (-22.8 - 38.1) | 0.849 | 0.430 |
| VNA classification (n) | | | | | | |
|  | Type I (35) | 7 (20.6) | 12 (22.2) | 16 (25) | 1.630 | 0.443 |
|  | Type II (42) | 8 (23.5) | 14 (25.9) | 20 (31.2) |  |  |
|  | Type III (28) | 5 (14.7) | 12 (22.2) | 11 (17.2) |  |  |
|  | Type IV (47) | 14 (41.2) | 16 (29.7) | 17 (26.6) |  |  |
| Cortical comminution in axial plane | | | | | | |
|  | Anterior (n, %) | 12 (35.3) | 15 (27.8) | 22 (34.4) | 0.766 | 0.682 |
|  | Anterior length (mm) | 10.8 ± 3.0 (6.87 - 16.2) | 10.3 ± 6.2 (4.4 - 28.7) | 9.6 ± 4.3 (4.2 - 20.9) | 0.289 | 0.751 |
|  | Posterior (n, %) | 28 (82.4) | 35 (64.8) | 41 (64.1) | 3.916 | 0.141 |
|  | Posterior length (mm) | 14.9 ± 5.2 (8.2 - 28.5) | 13.3 ± 4.4 (1.6 - 20.8) | 13.5 ± 4.0 (5.9 - 24.2) | 1.085 | 0.342 |
| Cortical comminution in coronal plane | | | | | | |
|  | Superior (n, %) | 20 (58.8) | 17 (31.5) | 27 (42.2) | 6.357 | 0.042 |
|  | Superior length (mm) | 11.5 ± 4.9 (2.56 - 22.0) | 11.1 ± 2.8 (7.4 - 18.8) | 11.0 ± 4.1 (4.6 - 19.2) | 0.079 | 0.924 |
|  | Inferior (n, %) | 25 (73.5) | 30 (55.6) | 38 (59.4) | 2.971 | 0.226 |
|  | Inferior length (mm) | 13.5 ± 5.2 (6.4 - 26.0) | 13.0 ± 4.6 (4.3 - 20.5) | 13.1 ± 6.1 (4.0 - 27.9) | 0.057 | 0.945 |
| Intraosseous defection (n, %) |  | 22 (64.7) | 27 (50) | 30 (46.9) | 2.939 | 0.230 |

**Supplement 4**. Gender differences with NSA, MPA, VNA and AMA.

| Variables | Male (n=78) | Female (n=74) | t | p |
| --- | --- | --- | --- | --- |
| NSA | 138.46 ± 5.68 | 140.49 ± 6.69 | 2.023 | 0.045 |
| MPA | 52.68 ± 9.85 | 46.73 ± 10.53 | 3.595 | 0.0001 |
| VNA | 11.10 ± 11.80 | 6.69 ± 12.14 | 2.268 | 0.025 |
| AMA | 94.23 ± 12.3 | 91.90 ± 12.10 | 1.052 | 0.294 |

**Supplement 5.** The distribution of anatomical, Pauwels, and VNA classifications and AMA groups with respect to key demographic characteristics.

| Classification or Groups | | Age (yrs) | F/t | p |  | Gender (n, %) | | χ2 | p |  | Side (n, %) | | χ2 | p |
| --- | --- | --- | --- | --- | --- | --- | --- | --- | --- | --- | --- | --- | --- | --- |
|  |  |  |  |  |  | Male (n=78) | Female (n=74) |  |  |  | Left (n=73) | Right (n=79) |  |  |
| Anatomical classification (n) | | | | | | | | | | | | | | |
|  | Subcapital (23) | 55.17 ± 16.38 | 0.299 | 0.742 |  | 5 (21.7) | 18 (78.3) | 10.01 | 0.007 |  | 14 (60.9) | 9 (39.1) | 1.83 | 0.401 |
|  | Transcervical (125) | 52.75 ± 14.66 |  |  |  | 71 (56.8) | 54 (43.2) |  |  |  | 57 (45.6) | 68 (54.4) |  |  |
|  | Basicervical (4) | 55.25 ± 8.18 |  |  |  | 2 (50.0) | 2 (50.0) |  |  |  | 2 (50.0) | 2 (50.0) |  |  |
| Pauwels classification (n) | | | | | | | | | | | | | | |
|  | Type I (8) | 60.50 ± 16.69 | 1.260 | 0.287 |  | 3 (37.5) | 5 (62.5) | 10.548 | 0.005 |  | 4 (50.0) | 4 (50.0) | 1.628 | 0.443 |
|  | Type II (60) | 51.82 ± 17.21 |  |  |  | 22 (36.7) | 38 (63.3) |  |  |  | 25 (41.7) | 35 (58.3) |  |  |
|  | Type III (84) | 53.46 ± 12.47 |  |  |  | 53 (63.1) | 31 (36.9) |  |  |  | 44 (52.4) | 40 (47.6) |  |  |
| VNA classification (n) | | | | | | | | | | | | | | |
|  | Type I (35) | 54.20 ± 18.13 | 0.682 | 0.564 |  | 11 (31.4) | 24 (68.6) | 11.012 | 0.012 |  | 15 (42.9) | 20 (57.1) | 0.738 | 0.864 |
|  | Type II (42) | 50.62 ± 14.77 |  |  |  | 20 (47.6) | 22 (52.4) |  |  |  | 20 (47.6) | 22 (52.4) |  |  |
|  | Type III (28) | 52.93 ± 13.06 |  |  |  | 20 (71.4) | 8 (28.6) |  |  |  | 15 (53.6) | 13 (46.4) |  |  |
|  | Type IV (47) | 54.87 ± 12.94 |  |  |  | 27 (57.4) | 20 (42.6) |  |  |  | 23 (48.9) | 24 (51.1) |  |  |
| AMA groups (n, %) | | | | | | | | | | | | | | |
|  | Group I (34) | 51.53 ± 12.64 | 1.325 | 0.269 |  | 15(44.1) | 19(55.9) | 1.003 | 0.606 |  | 18(52.9) | 16(47.1) | 0.428 | 0.807 |
|  | Group II (54) | 53.41 ± 10.72 |  |  |  | 28(51.9) | 26(48.1) |  |  |  | 25(46.3) | 29(53.7) |  |  |
|  | Group III (64) | 49.61 ± 14.05 |  |  |  | 35(54.7) | 29(45.3) |  |  |  | 30(46.9) | 34(53.1) |  |  |

**Supplement 6.** The distribution of MPA, VNA, Pauwels classification, and VNA classification among three anatomical fracture types

| Variables | | Subcapital (23) | Transcervical (125) | Basicervical (4) | F/χ2 | p |
| --- | --- | --- | --- | --- | --- | --- |
| MPA (°) |  | 40.86 ± 9.31 | 51.25 ± 10.15 | 55.18 ± 3.06 | 11.224 | <0.001 |
| Pauwels classification (n) | | | | | | |
|  | Type I (8) | 4 (17.4) | 4 (3.2) | 0 (0) | 31.371 | <0.001 |
|  | Type II (60) | 17 (73.9) | 43 (34.4) | 0 (0) |  |  |
|  | Type III (84) | 2 (8.7) | 78 (62.4) | 4 (100) |  |  |
| VNA (°) |  | -1.88 ± 10.08 | 10.60 ± 11.52 | 19.90 ± 3.16 | 13.985 | <0.001 |
| VNA classification (n) | | | | | | |
|  | Type I (35) | 13 (56.5) | 22 (17.6) | 0 (0) | 28.301 | <0.001 |
|  | Type II (42) | 6 (26.1) | 36 (28.8) | 0 (0) |  |  |
|  | Type III (28) | 3 (13.0) | 25 (20.0) | 0 (0) |  |  |
|  | Type IV (47) | 1 (4.3) | 42 (33.6) | 4 (100) |  |  |

**Supplement 7.** Literature review from 2011-2022 of the morphological details of the fracture models in physical biomechanical studies focusing on FNFs in young population

| First author | Year of Publication | Fracture classification | Fracture angle | | Cortical comminution | Characteristics of comminution simulation |
| --- | --- | --- | --- | --- | --- | --- |
|  |  |  | in coronal plane | in axial plane* |  |  |
| Liu JC [1] | 2022 | Subcapital and Pauwels | 60° | 90° | No | No |
| Steffensmeier A [2] | 2021 | OTA/AO 31-B2.3 and Pauwels | III | 90° | No | No |
| Jiang DJ [3] | 2021 | Transcervical and Pauwels | 70° | 90° | No | No |
| Gao ZC [4] | 2021 | Transcervical and Pauwels | 70° | 90° | No | No |
| Zhang YL [5] | 2020 | Transcervical and Pauwels | 80° | 90° | No | No |
| Wright DJ [6] | 2020 | Transcervical and Pauwels | 90° | 90°, 114° | Yes | 15° Posterior wedge osteotomy in the proximal fragment, with apex located half way between the superior and inferior borders |
| Schopper C [7] | 2020 | Transcervical and Pauwels | 40° | 90° | Yes | A 15° dorsally located wedge osteotomy |
| Nwankwo CD [8] | 2020 | OTA/AO 31-B2.3 or Transcervical and Pauwels | 70° | 90° | No | No |
| Brattgjerd JE [9] | 2020 | Transcervical and Pauwels | 55° | 90° | Yes | A 18° subcapitcal inferior wedge osteotomy with a maximum width of 7.5mm in the frontal plane |
| Bliven E [10] | 2020 | OTA/AO 31B2 and Pauwels | 70° | 90° | Yes | A calcar wedge and smaller posterior wedge osteotomy positioned cranial dorsal of the femoral neck |
| Liu JW [11] | 2019 | Transcervical and Pauwels | 70° | 90° | Yes | A distal 30° wedge and a posterior 15° wedge osteotomy |
| Kuan FC [12] | 2019 | Pauwels | 80° | 90° | No | No |
| Knobe M [13] | 2019 | OTA/AO 31-B2 and Pauwels | 70° | Unknown | Yes | A distal 30° wedge and a posterior 15° wedge osteotomy |
| Johnson J [14] | 2018 | Basicervical and Pauwels | II | 90° | No | No |
| Jafarov A [15] | 2019 | Transcervical and Pauwels | 70° | 90° | No | No |
| Giordano V [16] | 2019 | Transcervical and Pauwels | 70° | 90° | No | No |
| Zhang BK [17] | 2018 | Transcervical and VN | 20° | 90° | No | No |
| Zhang BK [18] | 2018 | Basicervical and Pauwels | 50°60°70° | 90° | No | No |
| Mansur H [19] | 2018 | Transcervical and Pauwels | II | Unknown | No | No |
| Magone KM [20] | 2018 | Transcervical and Pauwels | 62° | 90 | No | No |
| Knobe M [21] | 2017 | OTA/AO 31-B2 or Transcervical and Pauwels | 70° | 90° | Yes | A distal 30° wedge and a posterior 15° wedge osteotomy |
| Brattgjerd JE [22] | 2018 | Transcervical | Unknown | 90° | No | No |
| Stoffel K [23] | 2017 | OTA/AO 31-B2.3 and Pauwels | 70° | 90° | Yes | A distal 30° wedge and a posterior 15° wedge osteotomy |
| Kemker B [24] | 2017 | Basicervical and Pauwels | 62.5° | 90° | No | No |
| Johnson JP [25] | 2017 | Transcervical and Pauwels | 90° | 90° | No | No |
| Samsami S [26] | 2016 | Transcervical and Pauwels | 70° | 90° | No | No |
| Kuan FC [27] | 2016 | Transcervical and Pauwels | 70° | 90° | No | No |
| Schaefer TK [28] | 2015 | Subcapital | Unknown | 90° | Yes | A 1 cm bone block out of the posterior neck cut |
| Samsami S [29] | 2015 | Transcervical and Pauwels | 70° | 90° | No | No |
| Kunapuli SC [30] | 2015 | OTA/AO 31-B2.3 and Pauwels | 70° | 90° | No | No |
| Imren Y [31] | 2015 | Basicervical and Pauwels | 70° | 90° | No | No |
| Gardner A [32] | 2015 | OTA/AO 31-B1 valgus and Pauwels | 55°-63° | Unknown | No | No |
| Filipov O [33] | 2015 | OTA/AO 31-B2.2 or subcapital and Pauwels | II | 90° | No | No |
| Saglam N [34] | 2014 | Transcervical and Pauwels | 85° | Unknown | No | No |
| Freitas A [35-38] | 2014 | Transcervical and Pauwels | 70° | 90° | No | No |
| Basso T [39-41] | 2014 | Subcapital and Pauwels | 60° | 90° | No | No |
| Hawks MA [42] | 2013 | Transcervical and Pauwels | 90° | 90° | No | No |
| Nowotarski PJ [43] | 2012 | Transcervical and Pauwels | 80° | 90° | No | No |
| Zhang Y [44] | 2011 | Pauwels | 50° | 90° | No | No |
| Rupprecht M [45] | 2011 | Transcervical and Pauwels | 70° | 90° | No | No |

Only studies that met the following criteria were considered eligible: biomechanical study or experiment; Humans species; models simulated FNFs in young population; single fracture rather than composite fracture; fracture treated with any type of internal fixation; authors reported original research; only English language articles. * The angle is the anteromedial oblique angle formed by the fracture line and the centerline of femoral neck in the axial plane. OTA/AO, Orthopaedic Trauma Association/ Arbeitsgemeinschaftfür Osteosynthesefragen. VN, vertical of the neck axis.

**References**

1. Liu J, Li Z, Ding J, Huang B, Piao C et al. Biomechanical analysis of two medial buttress plate fixation methods to treat Pauwels type III femoral neck fractures. BMC musculoskeletal disorders 2022;23:49-55.

2. Steffensmeier A, Shah N, Archdeacon M, Watson D, Sanders RW, et al. et al. Clinical and Biomechanical Effects of Femoral Neck Buttress Plate used for Vertical Femoral Neck Fractures. Injury 2021;53:1137-1142.

3. Jiang D, Zhan S, Wang L, Shi LL, Ling M, et al. et al. Biomechanical comparison of five cannulated screw fixation strategies for young vertical femoral neck fractures. J Orthop Res 2021;39:1669-1680.

4. Gao Z, Wang M, Shen B, Chu X, Ruan D et al. Treatment of Pauwels type III femoral neck fracture with medial femoral neck support screw: a biomechanical and clinical study. Scientific reports 2021;11:21418.

5. Zhang Y, Yan C, Zhang L, Zhang W, Wang G et al. Comparison of Ordinary Cannulated Compression Screw and Double-Head Cannulated Compression Screw Fixation in Vertical Femoral Neck Fractures. BioMed research international 2020;2020:2814548.

6. Wright DJ, Bui CN, Ihn HE, McGarry MH, Lee TQ, et al. et al. Posterior Inferior Comminution Significantly Influences Torque to Failure in Vertically Oriented Femoral Neck Fractures: A Biomechanical Study. Journal of orthopaedic trauma 2020;34:644-649.

7. Schopper C, Zderic I, Menze J, Müller D, Rocci M, et al. et al. Higher stability and more predictive fixation with the Femoral Neck System versus Hansson Pins in femoral neck fractures Pauwels II. Journal of orthopaedic translation 2020;24:88-95.

8. Nwankwo CD, Schimoler P, Greco V, Kharlamov A, Westrick ER, et al. et al. Medial Plating of Pauwels Type III Femoral Neck Fractures Decreases Shear and Angular Displacement Compared with a Derotational Screw. Journal of orthopaedic trauma 2020;34:639-643.

9. Brattgjerd JE, Steen H, Strømsøe K et al. Increased stability by a novel femoral neck interlocking plate compared to conventional fixation methods. A biomechanical study in synthetic bone. Clinical biomechanics (Bristol, Avon) 2020;76:104995.

10. Bliven E, Sandriesser S, Augat P, von Rüden C, Hackl S et al. Biomechanical evaluation of locked plating fixation for unstable femoral neck fractures. Bone & joint research 2020;9:314-321.

11. Liu J, Zhang B, Yin B, Chen H, Sun H, et al. et al. Biomechanical Evaluation of the Modified Cannulated Screws Fixation of Unstable Femoral Neck Fracture with Comminuted Posteromedial Cortex. BioMed research international 2019;2019:2584151.

12. Kuan FC, Hsu KL, Lin CL, Hong CK, Yeh ML, et al. et al. Biomechanical properties of off-axis screw in Pauwels III femoral neck fracture fixation: Bicortical screw construct is superior to unicortical screw construct. Injury 2019;50:1889-1894.

13. Knobe M, Bettag S, Kammerlander C, Altgassen S, Maier KJ, et al. et al. Is bone-cement augmentation of screw-anchor fixation systems superior in unstable femoral neck fractures? A biomechanical cadaveric study. Injury 2019;50:292-300.

14. Johnson J, Deren M, Chambers A, Cassidy D, Koruprolu S, et al. et al. Biomechanical Analysis of Fixation Devices for Basicervical Femoral Neck Fractures. The Journal of the American Academy of Orthopaedic Surgeons 2019;27:e41-e48.

15. Jafarov A, Erbay Elibol FK, Alizadeh C, Gülşen M, Toğrul E, et al. et al. Perforated H-beam implant can be used in femoral neck fracture. Proceedings of the Institution of Mechanical Engineers Part H, Journal of engineering in medicine 2019;233:354-361.

16. Giordano V, Alves DD, Paes RP, Amaral AB, Giordano M, et al. et al. The role of the medial plate for Pauwels type III femoral neck fracture: a comparative mechanical study using two fixations with cannulated screws. Journal of experimental orthopaedics 2019;6:18-26.

17. Zhang B, Liu J, Zhu Y, Zhang W et al. A new configuration of cannulated screw fixation in the treatment of vertical femoral neck fractures. International orthopaedics 2018;42:1949-1955.

18. Zhang B, Liu J, Zhang W et al. Ordinary Cannulated Compression Screws or Headless Cannulated Compression Screws? A Synthetic Bone Biomechanical Research in the Internal Fixation of Vertical Femoral Neck Fracture. BioMed research international 2018;2018:4898301.

19. Mansur H, Alvarez R, Freitas A, Gonçalves CB, Ramos MRF et al. BIOMECHANICAL ANALYSIS OF FEMORAL NECK FRACTURE FIXATION IN SYNTHETIC BONE. Acta ortopedica brasileira 2018;26:162-165.

20. Magone KM, Owen JK, Kemker BP, Bloom O, Martin S, et al. et al. A model to evaluate Pauwels type III femoral neck fractures. Proceedings of the Institution of Mechanical Engineers Part H, Journal of engineering in medicine 2018;232:310-317.

21. Knobe M, Altgassen S, Maier KJ, Gradl-Dietsch G, Kaczmarek C, et al. et al. Screw-blade fixation systems in Pauwels three femoral neck fractures: a biomechanical evaluation. International orthopaedics 2018;42:409-418.

22. Brattgjerd JE, Loferer M, Niratisairak S, Steen H, Stromsoe K et al. Increased torsional stability by a novel femoral neck locking plate. The role of plate design and pin configuration in a synthetic bone block model. Clinical biomechanics (Bristol, Avon) 2018;55:28-35.

23. Stoffel K, Zderic I, Gras F, Sommer C, Eberli U, et al. et al. Biomechanical Evaluation of the Femoral Neck System in Unstable Pauwels III Femoral Neck Fractures: A Comparison with the Dynamic Hip Screw and Cannulated Screws. Journal of orthopaedic trauma 2017;31:131-137.

24. Kemker B, Magone K, Owen J, Atkinson P, Martin S, et al. et al. A sliding hip screw augmented with 2 screws is biomechanically similar to an inverted triad of cannulated screws in repair of a Pauwels type-III fracture. Injury 2017;48:1743-1748.

25. Johnson JP, Borenstein TR, Waryasz GR, Klinge SA, McClure PK, et al. et al. vertically oriented femoral neck fractures: a biomechanical comparison of 3 fixation constructs. Journal of orthopaedic trauma 2017;31:363-368.

26. Samsami S, Saberi S, Bagheri N, Rouhi G et al. Interfragmentary motion assessment for three different fixation techniques of femoral neck fractures in young adults. Bio-medical materials and engineering 2016;27:389-404.

27. Kuan FC, Yeh ML, Hong CK, Chiang FL, Jou IM, et al. et al. Augmentation by cerclage wire improves fixation of vertical shear femoral neck fractures-A biomechanical analysis. Injury 2016;47:2081-2086.

28. Schaefer TK, Spross C, Stoffel KK, Yates PJ et al. Biomechanical properties of a posterior fully threaded positioning screw for cannulated screw fixation of displaced neck of femur fractures. Injury 2015;46:2130-2133.

29. Samsami S, Saberi S, Sadighi S, Rouhi G et al. Comparison of Three Fixation Methods for Femoral Neck Fracture in Young Adults: Experimental and Numerical Investigations. Journal of medical and biological engineering 2015;35:566-579.

30. Kunapuli SC, Schramski MJ, Lee AS, Popovich JM, Jr., Cholewicki J, et al. et al. Biomechanical analysis of augmented plate fixation for the treatment of vertical shear femoral neck fractures. Journal of orthopaedic trauma 2015;29:144-150.

31. Imren Y, Gurkan V, Bilsel K, Desteli EE, Tuna M, et al. et al. Biomechanical comparison of dynamic hip screw, proximal femoral nail, cannulated screw, and monoaxial external fixation in the treatment of basicervical femoral neck fractures. Acta chirurgiae orthopaedicae et traumatologiae Cechoslovaca 2015;82:140-144.

32. Gardner AW, Toh MZ, Yew KS, Lie DT, Chou SM et al. Cannulated versus non-cannulated cancellous screw fixation for femoral neck fractures: a synthetic bone biomechanical study. Journal of orthopaedic surgery (Hong Kong) 2015;23:41-46.

33. Filipov O, Gueorguiev B et al. Unique stability of femoral neck fractures treated with the novel biplane double-supported screw fixation method: a biomechanical cadaver study. Injury 2015;46:218-226.

34. Saglam N, Kucukdurmaz F, Kivilcim H, Kurtulmus T, Sen C, et al. et al. Biomechanical comparison of antirotator compression hip screw and cannulated screw fixations in the femoral neck fractures. Acta Orthop Traumatol Turc 2014;48:196-201.

35. Freitas A, Torres GM, Souza AC, Maciel RA, Souto DR, et al. et al. Analysis on the mechanical resistance of fixation of femoral neck fractures in synthetic bone, using the dynamic hip system and an anti-rotation screw. Revista brasileira de ortopedia 2014;49:586-592.

36. Freitas A, Maciel RA, Lima Rde A, Souto DR, Ferrer Mde A et al. Mechanical analysis of femoral neck fracture fixation with dynamic condylar screw in synthetic bone. Acta ortopedica brasileira 2014;22:264-268.

37. Freitas A, Lula WF, de Oliveira JS, Maciel RA, Souto DR, et al. et al. Analysis of mechanical strength to fixing the femoral neck fracture in synthetic bone type Asnis. Acta ortopedica brasileira 2014;22:206-209.

38. Freitas A, Azevedo BA, de Souza RR, da Costa HI, Maciel RA, et al. et al. Mechanical analysis of femoral neck fracture fixation in synthetic bone. Acta Ortop Bras 2014;22:155-158.

39. Basso T, Klaksvik J, Syversen U, Foss OA et al. A biomechanical comparison of composite femurs and cadaver femurs used in experiments on operated hip fractures. Journal of biomechanics 2014;47:3898-3902.

40. Basso T, Klaksvik J, Foss OA et al. The effect of interlocking parallel screws in subcapital femoral-neck fracture fixation: a cadaver study. Clinical biomechanics (Bristol, Avon) 2014;29:213-217.

41. Basso T, Klaksvik J, Foss OA et al. Locking plates and their effects on healing conditions and stress distribution: A femoral neck fracture study in cadavers. Clinical biomechanics (Bristol, Avon) 2014;29:595-598.

42. Hawks MA, Kim H, Strauss JE, Oliphant BW, Golden RD, et al. et al. Does a trochanteric lag screw improve fixation of vertically oriented femoral neck fractures? A biomechanical analysis in cadaveric bone. Clinical biomechanics (Bristol, Avon) 2013;28:886-891.

43. Nowotarski PJ, Ervin B, Weatherby B, Pettit J, Goulet R, et al. et al. Biomechanical analysis of a novel femoral neck locking plate for treatment of vertical shear Pauwel's type C femoral neck fractures. Injury 2012;43:802-806.

44. Zhang Y, Tian L, Yan Y, Sang H, Ma Z, et al. et al. Biomechanical evaluation of the expansive cannulated screw for fixation of femoral neck fractures. Injury 2011;42:1372-1376.

45. Rupprecht M, Grossterlinden L, Ruecker AH, de Oliveira AN, Sellenschloh K, et al. et al. A comparative biomechanical analysis of fixation devices for unstable femoral neck fractures: the Intertan versus cannulated screws or a dynamic hip screw. The Journal of trauma 2011;71:625-634.
